# Spontaneous mesenteric hematoma complicating an exacerbation of Crohn’s disease: report of a case

**DOI:** 10.1186/1471-2482-14-35

**Published:** 2014-06-03

**Authors:** Hutan Ashrafian, James HR Manfield, Anuja Mitra, Derek J Boyle, Pawan Mathur

**Affiliations:** 1 Department of General Surgery, Barnet Hospital, Barnet and Chase Farm Hospitals NHS Trust, Wellhouse Lane, Barnet EN5 3DJ, UK; 2 Department of Surgery and Cancer, Imperial College London, 10th Floor QEQM Building, St Mary’s Hospital, Praed Street, London W2 1NY, UK

**Keywords:** Crohn’s disease, Mesenteric haematoma, Inflammatory bowel disease, Surgery

## Abstract

**Case Presentation:**

Spontaneous mesenteric haematoma is a rare condition that occurs due to localized bleeding in the mesenteric vascular tree of a bowel segment in the absence of an identifiable cause. Here we report a case of spontaneous mesenteric haematoma during an inflammatory exacerbation of Crohn’s disease. The patient underwent surgical management for small bowel obstruction secondary to Crohn’s disease, however the concurrent presence of a spontaneous mesenteric haematoma in the mid-jejunal mesentery was successfully managed conservatively.

**Conclusion:**

This case identifies the first association of spontaneous mesenteric haematoma with an exacerbation of Crohn’s disease and highlights the need to consider rare differential diagnoses such as SMH when performing radiological assessment of unexplained symptoms in inflammatory bowel disease patients.

## Background

Spontaneous mesenteric haematoma (SMH) is a rare condition of unknown aetiology. It can be managed conservatively in the event that there is no associated mesenteric haemorrhage. It must be clinically distinguished from spontaneous mesenteric intraperitoneal haemorrhage (or abdominal apoplexy) where there is rupture or bleeding from a specific mesenteric vessel due to an unknown cause that typically requires urgent surgical management due to its high mortality. This report presents the first case in the literature associating SMH with an inflammatory exacerbation of Crohn’s disease.

### Case presentation

**Figure 1:**
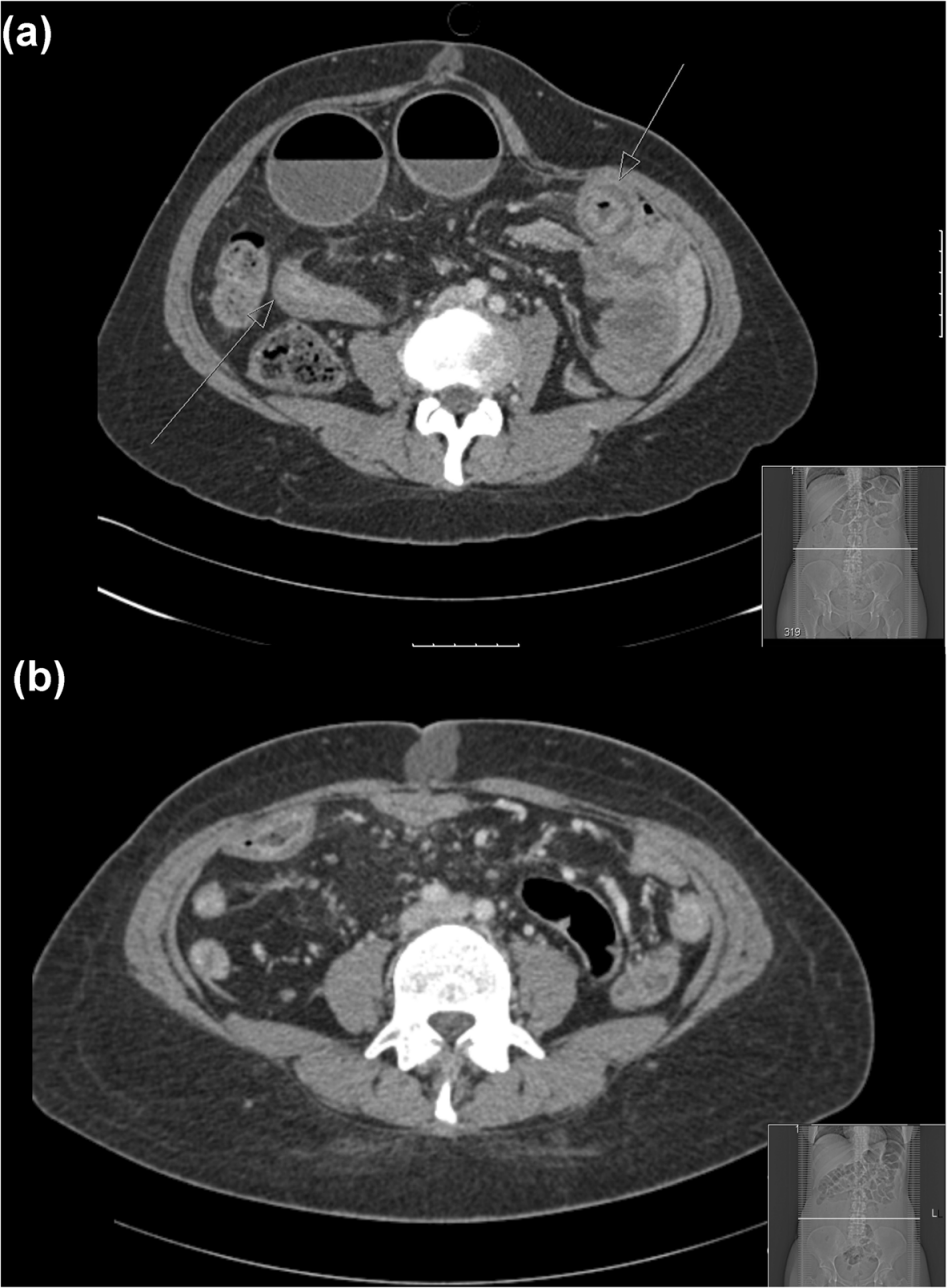
Initial computed tomography (CT) scan demonstrating (a) Crohn’s strictures; (b) Absence of mesenteric haematoma.

**Figure 2:**
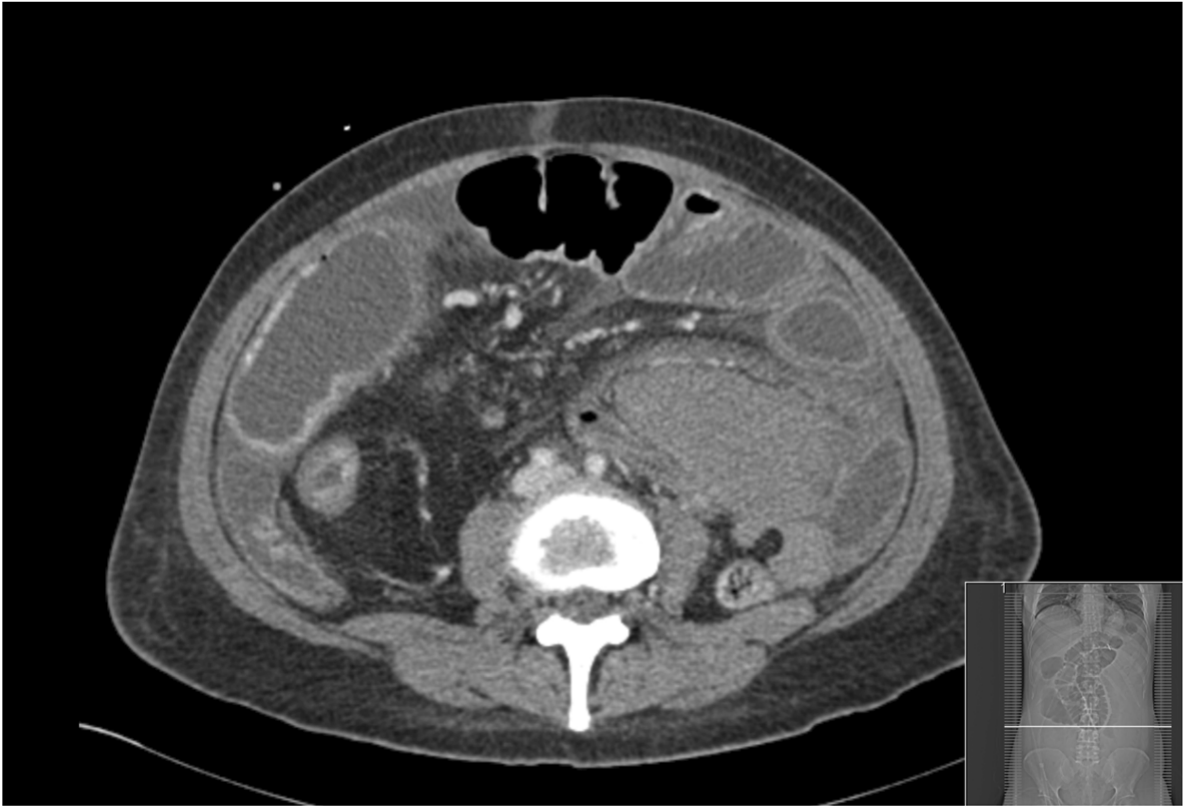
**Computed tomography (CT) scan demonstrating small bowel obstruction and mesenteric haematoma taken at the same anatomical level as Figure**1**b.**

A 44-year-old female presented with an exacerbation of Crohn’s disease that was not responsive to medical management with escalating steroid (prednisolone) and purine analogue (azathioprine) therapy. She was a non-smoker and aside from well-controlled essential hypertension had no other significant co-morbidities. Her inflammatory bowel disease had already required two previous hospitalizations, and she had suffered from symptoms of intermittent pain and bloating with constipation throughout the 12 months since her diagnosis. In the preceding month a computed tomography (CT) scan revealed 3 segments of Crohn’s disease strictures with prestenotic dilatation and an inflammatory appearance (Figure 1a). No mesenteric haematoma was identified at this point (Figure 1b). Blood tests taken at this time were unremarkable with a haemaglobin (Hb) of 11.7 g/dl, white cell count (WCC) of 5.4 × 10^9^/L and a C-reactive protein (CRP) of 7 mg/L. Her symptoms initially resolved with intravenous hydrocortisone but promptly recurred with worsening malnutrition despite intensifying steroid therapy. She had not received any formal anticoagulation other than prophylactic subcutaneous low-molecular weight heparin (20 mg once daily) during the period of her hospitalization. Repeat CT scan (Figure 2) demonstrated a significant deterioration in small bowel dilatation with impending obstruction due to a 10 cm distal ileal stricture and a left upper quadrant abdominal ‘mass’ (9 × 12 × 20 cm), thought to be an inflammatory phlegmon secondary to an area of more proximal Crohn’s disease. Repeat blood tests at this time revealed anaemia with a haemaglobin of 8.6 g/dl, a normal WCC of 6.5 × 10^9^/L and a CRP of 25 mg/L. No clotting abnormalities were identified and liver function tests were unremarkable.

**Figure 3:**
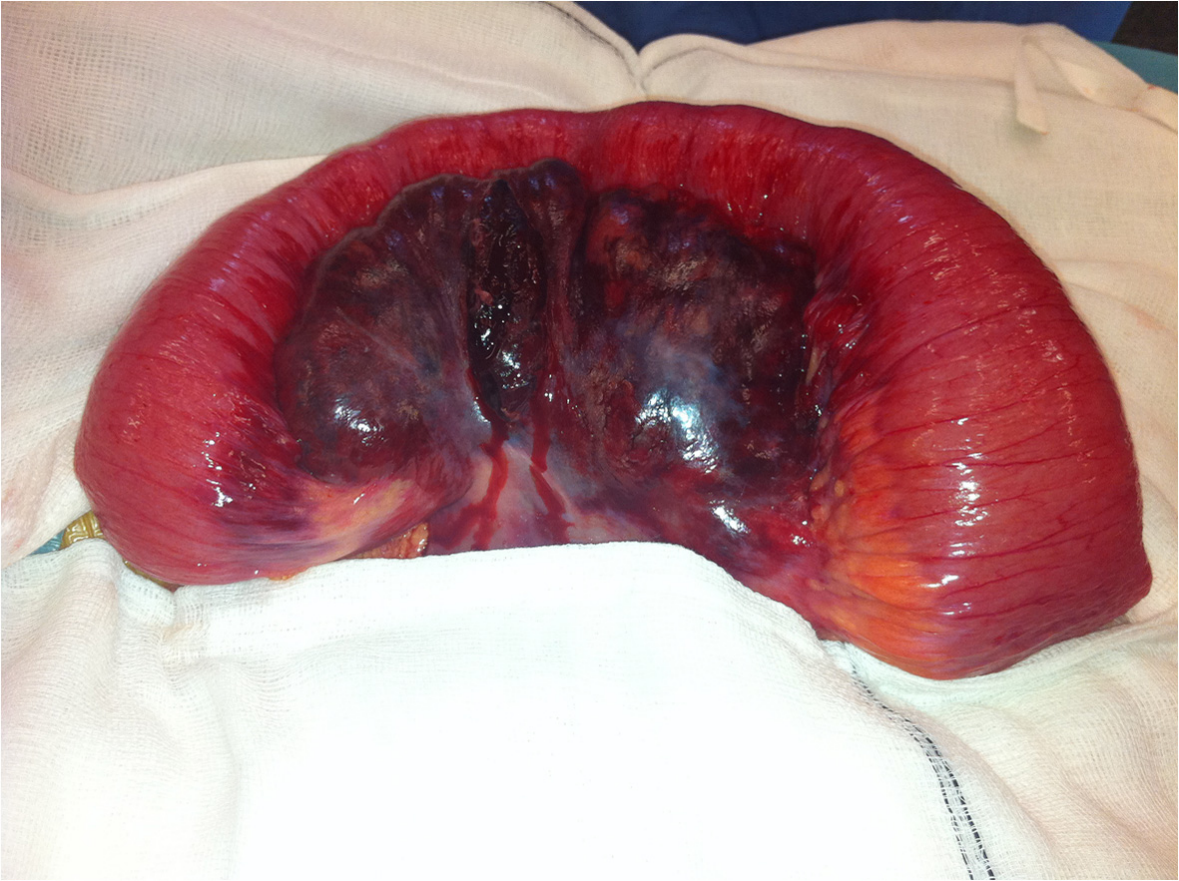
Intraoperative image of mid-jejunal mesenteric haematoma.

Subsequent laparotomy identified a grossly dilated segment of ileum proximal to a terminal ileal stricture. There was also a large haematoma within the mesentery of the mid-jejunum that corresponded to the mass demonstrated on CT (Figure 3). There was no evidence of local vascular trauma or pancreatico-biliary inflammation. The loop of jejenum was viable and macroscopically normal without any associated signs of Crohn’s disease. The remainder of the small and large bowel also appeared normal. The patient underwent a limited right hemicolectomy and double barrel stoma formation from the ileum and ascending colon. After surgery she was placed on an enhanced recovery protocol and was discharged on the 5^th^ post-operative day. She is currently well and to date her follow-up has remained un-eventful.

## Discussion

The surgical complications of Crohn’s disease include bowel obstruction, fistulation, ulceration, anal fissures and cancer [1]. The pathological severity of this condition derives from a combination of inherited factors and environmental triggers that lead to gut inflammation and local microvascular ischaemia [2,3]. The vascular component of Crohn’s disease has been primarily associated with arterial and venous thrombotic events that can lead to mesenteric ischaemia, venous congestion and bowel perforation [1,4]. There is an association between patients with Crohn’s disease who suffer from intramural bowel haematomas, bleeding diatheses and anticoagulation therapy [5]. To the best of our knowledge, we report the first case in the literature of spontaneous jejunal mesenteric (mesojejunal) haematoma.

Spontaneous mesenteric haematoma (SMH) is a rare condition that occurs due to localized bleeding in the mesenteric vascular tree of a bowel segment with unknown aetiology [6-8]. It can be clinically distinguished from the related disease pathology of spontaneous mesenteric intraperitoneal haemorrhage (abdominal apoplexy) that occurs due to the spontaneous rupture of a specific mesenteric vessel likely from undiagnosed mesenteric arterial dissection and perivascular pseudo aneurysms. In these cases there is evidence of both intraperitoneal and retroperitoneal haemorrhage [9]. SMH can be managed conservatively [6-8] if there is no associated haemorrhage and although the patient in this case underwent surgery for intestinal stenosis, the concurrent mesenteric haematoma was successfully managed conservatively.

Patients with isolated SMH typically present with non-specific symptoms of generalised abdominal pain and as a result the haematoma is usually identified on imaging modalities such as CT. Although the cause is unknown, the disease process may be associated with unidentified anticoagulation mismanagement or even undiagnosed connective tissue disease [6-8].

Our case demonstrates the presence of localised mid-jejunal mesenteric fat haematoma. Localised visceral inflammation is a recognised contributor to confined microvascular bleeding [10] so that the inflammatory exacerbation of Crohn’s disease in this case may have resulted in severe mesenteric fat inflammation to initiate localised capillary leakage and subsequent haematoma.

Mesenteric fat has been recently recognised an essential location for the generation of Crohn’s gastrointestinal inflammation [11,12]. Here bacterial translocation associated with immunoregulation through nucleotide-binding oligomerization domains (NOD1 and 2) and Toll-like receptors (TLRs 2 and 4) result in an increase in local inflammatory mediators including C-reactive-protein (CRP). The powerful inflammatory role of the bowel mesentery might be a contributory factor in the association between Crohn’s exacerbation and mesenteric haematoma in this case.

Our intraoperative findings of mesojejunal haematoma reflect the current underestimation of Crohn’s ileitis, demonstrating explicit involvement of the mesenteric fat with an associated mesenteric haematoma. This case also highlights how mesenteric abnormalities such as SMH may explain refractoriness or recurrence of symptoms despite intensifying steroid therapy. Indeed, it is possible that ileal compression caused by a mesenteric mass such as this may explain many of the symptoms accredited to Crohn’s strictures. As a result, we believe that an increased awareness of the role of the bowel mesentery may give new insights into Crohn’s disease pathogenesis. Furthermore, assessment for mesenteric abnormalities may guide future clinical and radiological assessment of unexplained symptoms in patients suffering form this multi-systemic inflammatory process, and identify rare differential diagnoses such as SMH.

## Conclusion

We describe a case of spontaneous mesenteric haematoma in a female patient suffering from an exacerbation of Crohn’s disease and intestinal stenosis. The mesenteric haematoma was identifiable both on pre-operative computerised tomography and intra-operatively. This case identifies the first association of spontaneous mesenteric haematoma with an exacerbation of Crohn’s disease and highlights the need to consider rare differential diagnoses such as SMH when performing radiological assessment of unexplained symptoms in inflammatory bowel disease patients.

## Consent

Written informed consent was obtained from the patient for publication of this case report and any accompanying images. A copy of the written consent is available for review by the Editor of this journal.

## Abbreviations

CRP: C-reactive protein; TLR: Toll-like receptor; NOD: Nucleotide-binding oligomerization domain; SMH: Spontaneous mesenteric haematoma; CT: Computerized tomography.

## Competing interests

The author(s) declare that they have no competing interests.

## Authors’ contributions

PM carried out the procedure, assisted by HA and DB. HA, JM and AM drafted the manuscript. HA finalized the manuscript in agreement with the senior author PM. All authors read and approved the final manuscript.

## Pre-publication history

The pre-publication history for this paper can be accessed here:

http://www.biomedcentral.com/1471-2482/14/35/prepub

## References

[B1] Gardiner KR Dasari BV Operative management of small bowel Crohn’s disease Surg Clin North Am 2007 87 3 587 610 10.1016/j.suc.2007.03.011 17560414

[B2] Baumgart DC Sandborn WJ Crohn’s disease Lancet 2012 380 9853 1590 1605 10.1016/S0140-6736(12)60026-9 22914295

[B3] Thornton M Solomon MJ Crohn’s disease: in defense of a microvascular aetiology Int J Color Dis 2002 17 5 287 297 10.1007/s00384-002-0408-5 12172921

[B4] Ha C Magowan S Accortt NA Chen J Stone CD Risk of arterial thrombotic events in inflammatory bowel disease Am J Gastroenterol 2009 104 6 1445 1451 10.1038/ajg.2009.81 19491858

[B5] Abbas MA Collins JM Olden KW Spontaneous intramural small-bowel hematoma: imaging findings and outcome AJR Am J Roentgenol 2002 179 6 1389 1394 10.2214/ajr.179.6.1791389 12438021

[B6] Parker SG Thompson JN Spontaneous mesenteric haematoma; diagnosis and management BMJ Case Rep 2012 2012 doi:10.1136/bcr-2012-006624. PMID: 22865811 10.1136/bcr-2012-006624 PMC4543710 22865811

[B7] Gomez D Rahman SH Guillou PJ Spontaneous mesenteric haematoma: a diagnostic challenge (On-line case report) Ann R Coll Surg Engl 2006 88 1 316884606

[B8] Ashley S Spontaneous mesenteric haematoma and small bowel infarction complicating oral anticoagulant therapy J R Soc Med 1990 83 2 116 2319537 10.1177/014107689008300221 PMC1292513

[B9] Cawyer JC Stone CK Abdominal apoplexy: a case report and review Am J Emerg Med 2011 40 3 e49 e52 10.1016/j.jemermed.2007.11.080 18687563

[B10] Whitcomb DC Muddana V Langmead CJ Houghton FD Jr Guenther A Eagon PK Mayerle J Aghdassi AA Weiss FU Evans A Lamb J Clermont G Lerch MM Papachristou GI Angiopoietin-2, a regulator of vascular permeability in inflammation, is associated with persistent organ failure in patients with acute pancreatitis from the United States and Germany AmJ Gastroenterol 2010 105 10 2287 2292 10.1038/ajg.2010.183 20461065

[B11] Peyrin-Biroulet L Gonzalez F Dubuquoy L Rousseaux C Dubuquoy C Decourcelle C Saudemont A Tachon M Beclin E Odou MF Neut C Colombel JF Desreumaux P Mesenteric fat as a source of C reactive protein and as a target for bacterial translocation in Crohn’s disease Gut 2012 61 1 78 85 10.1136/gutjnl-2011-300370 21940721 PMC3230831

[B12] Siegmund B Mesenteric fat in Crohn’s disease: the hot spot of inflammation? Gut 2012 61 1 3 5 10.1136/gutjnl-2011-301354 22068165

